# Cross one single body 49 tissues single-cell transcriptome reveals detailed macrophage heterogeneity during pig pregnancy

**DOI:** 10.3389/fimmu.2025.1574120

**Published:** 2025-04-02

**Authors:** Xiaoyun Chen, Chenliang Lai, Liping Cai, Lusheng Huang

**Affiliations:** National Key Laboratory for Swine Genetic Improvement and Germplasm Innovation, Ministry of Science and Technology of China, Jiangxi Agricultural University, Nanchang, China

**Keywords:** macrophages, pregnant pig, single-cell transcriptome, cross-tissue, functional heterogeneity, pregnancy-related changes, cross-species

## Abstract

**Introduction:**

Pregnancy involves complex physiological adaptations across maternal organs and the immune system to support fetal development. Macrophages play a dual role during pregnancy: defending against pathogens and supporting tissue adaptation. However, comprehensive and in-depth studies of cross-tissue transcriptional heterogeneity of macrophages during healthy pregnancy at the single-cell level remain elusive.

**Methods:**

We performed single-cell RNA sequencing (scRNA-seq) to profile macrophages from a healthy pregnant pig across 49 tissues. Immunofluorescence was performed to verify the specific expression of transcription factors.

**Results:**

In this study, we generated a macrophage atlas containing 114,881 macrophages from 49 tissues/organs within one single healthy pregnant pig, identified 33 subtypes, and revealed extensive tissue-specific diversity. We observed significant heterogeneity of macrophage subtypes across five different anatomical sites of adipose tissue. Notably, the Mφ MARCO+ subtype, primarily derived from mesenteric adipose tissue, showed higher activity in pattern recognition receptor signaling pathways compared to subtypes in other tissues, including different fat depots. Cross-tissue analysis revealed distinct expression patterns of transcription factors, cytokines, and cell surface receptors, including the transcription factor *PLSCR1*, specifically expressed in lung macrophages and verified by immunofluorescence. Cross-species analysis unveiled conservation and heterogeneity among macrophages in pigs, humans, and mice.

**Conclusion:**

We constructed a multiple-tissue single-cell transcriptome atlas of macrophages in one single healthy pregnant pig, revealing their molecular differences and commonalities across tissues and species. Our study provides a valuable resource for understanding macrophage diversity and tissue-specific macrophage adaptations during pregnancy in pigs.

## 1 Introduction

Macrophages (Mφ), a heterogeneous cell population, are distributed throughout the body and display remarkable diversity and plasticity, adapting their functions to the specific needs of various tissue environments (1–5). During embryonic development, macrophages colonize the entire embryo and differentiate into functionally and phenotypically distinct subtypes that persist throughout life (1, 6). For example, microglia in the central nervous system (CNS) mediate synaptic pruning and alter neuronal circuits (7, 8), and alveolar macrophages in the lung possess the ability to remove microbes, particles, and surfactants (9, 10). The transcription factors (TFs) play a crucial role in the fate diversification, identity maintenance, and functional regulation of macrophages (11, 12). Several tissue-specific TFs and their functions have been studied in detail (11). For instance, *ID3* not only acts as a lineage-determining for Kupffer cells but also mediates its role in restricting tumor growth (13), while *SPIC* regulates the development of red pulp macrophages and maintains iron homeostasis in the spleen (14).

Macrophages express a variety of receptors that enable them to sense niche signals from the surrounding microenvironment, including metabolites, extracellular matrix components, and molecular signals associated with apoptotic or damaged cells and pathogens, to guide appropriate tissue function and maintain tissue homeostasis (1, 15). Concretely, these receptors trigger macrophages to initiate tightly regulated signaling cascades that induce the production of various cytokines and other bioactive molecules to perform functions such as clearance of pathogens, cellular debris, and apoptotic cells, modulation of immune and inflammatory responses, extracellular matrix digestion and remodeling, and metabolic regulation (1, 3, 15). The complex signaling regulatory network between TFs, cell surface receptors, cytokines, and other genes is highly dependent on the environment and exhibits significant differences across tissues (16, 17). This complexity highlights the necessity for a comprehensive analysis of macrophage gene expression patterns in different tissues, laying the foundation for unraveling their diverse roles under various physiological conditions.

During pregnancy, the maternal body undergoes significant changes involving various organs and the immune system to support fetal growth and healthy development (18, 19). As essential immune and supportive cells within tissues (20), macrophages play a vital role in the physiological adaptations required during pregnancy (21–23). Researches on human decidual and placental macrophages have emphasized the dynamic changes in macrophage phenotype, polarity, and function that are necessary to meet distinct demands at different stages of pregnancy (21, 24–26). Specifically, human decidual macrophages are polarized to an immunosuppressive M2-like phenotype and secrete anti-inflammatory cytokines, acting as sentinels at the maternal-fetal interface and producing antimicrobial peptides while balancing tolerance to avoid rejection. Macrophages also clear apoptotic trophoblast cells and debris through efferocytosis, preventing inflammatory responses (24, 25, 27). In-depth studies of macrophages during normal pregnancy in pigs remain limited.

Pigs share striking similarities with humans in anatomical structure, physiology, immunology, and genomics, making them very suitable for biomedical research as model animals (28, 29). Comparative evaluation of genomes related to immune response reveals that porcine and human genomes and immune genes are more conserved relative to mice (30, 31). Specifically, the polarized responses of pig M1 macrophages to interferon-γ (IFN-γ) and lipopolysaccharide (LPS) predominantly exhibited a response similar to that of humans (30). Together, these findings strongly reinforce the view that pigs are a scientifically acceptable intermediate species between mice and humans, particularly for immunological research. Despite structural differences between pig and human placentas (32), pigs are also valuable models for studying the fundamental mechanisms of fetal medicine and obstetric disorders, including intrauterine growth restriction (33), and preeclampsia (34).

The inherent plasticity of tissue macrophages enables them to perform a wide range of functions in response to various physiological and pathological conditions, such as pregnancy, injury, and disease. Macrophages have been characterized in several organs in pigs using bulk RNA-seq or histology, revealing significant heterogeneity in their molecular phenotype and function (35). Advancements in single-cell RNA sequencing (scRNA-seq) in pigs have revolutionized our ability to explore novel cell types, dynamic changes of cell composition, and molecular heterogeneity of cell types within and among tissues (36–40). Recent studies on cross-tissue single-cell atlases have revealed that macrophages exhibit striking transcriptional heterogeneity across tissues under steady-state and developmental conditions (41, 42). By exploring the cross-tissue heterogeneity of pig macrophages, we can gain a better understanding of the complexity of the immune system and provide a reference for human macrophage research (43). Yet, a comprehensive and in-depth study of the compositional and molecular heterogeneity of macrophages across tissues in pigs, both in the normal and pregnant states, is still insufficient. Furthermore, controlled comparisons of cell types across tissues and organs are particularly challenging when donors differ in genetic background, age, environmental exposure, and epigenetic effects (44).

In this study, we constructed a comprehensive macrophage atlas of a healthy pregnant pig to uncover cell composition and gene expression characteristics of macrophages across tissues. In total, 114,881 macrophages from 54 tissues/organs passed quality control filtering and were clustered into 33 subtypes, revealing a remarkable diversity of tissue-specific macrophage subtypes. We provided a global view of macrophage heterogeneity through cross-tissue analysis for shared and tissue-specific macrophage subtypes, gene expression patterns, as well as immune and phagocytic functions. Focusing on the uterus, we revealed the characteristics of macrophages in both pregnant and non-pregnant states. Our findings preliminarily showed that macrophages may exhibit enhanced phagocytic activity and activated catabolic processes associated with tissue remodeling during pregnancy. We uncovered the conservation and heterogeneity between macrophages in pigs, humans, and mice through cross-species analysis. This study offers a valuable resource for understanding the diversity and functional heterogeneity of pig macrophages across tissues in the context of pregnancy.

## 2 Materials and methods

### 2.1 Animals

Pregnant sows, mated boars, and non-pregnant sows were Large White pigs from the Taihe County Aomu Breeding Co., Ltd. Pregnant sows at approximately 110 days of gestation were anesthetized with isoflurane before abdominal surgery and sample collection. All experiments involving the pigs were approved by the Animal Ethics Committee of Jiangxi Agricultural University (JXAULL-2021-37).

### 2.2 Tissue acquisition and processing, single-cell transcriptome sequencing and data analysis

#### 2.2.1 Tissue preparation

We sampled 113 anatomical regions of a single pregnant sow and 9 anatomical regions of a mated boar within about 30 minutes, divided into 54 tissues/organs. For detailed sample information, please refer to **Supplementary Table 1**. We also collected additional uterine horn tissue from a non-pregnant sow. Tissues designated for single-cell isolation were immediately placed on ice for further processing. For immunofluorescence staining, tissues were fixed in 10% neutral buffered formalin, with the fixative solution replaced after 48 hours to enhance tissue preservation.

#### 2.2.2 Tissue digestion

For single-cell isolation, nearly all tissues were subjected to enzymatic digestion using a cocktail (specify enzymes, e.g., collagenase, DNase, dispase. The enzyme combinations and their respective working concentrations for each tissue digestion solution were detailed in **Supplementary Table 2**) optimized for each tissue type. Briefly, the tissue was minced into small pieces and digested in a 37°C water bath with shaking at 110 rpm for 25-60 minutes, ensuring most tissue dissociation into a single cell. The cell suspension was filtered through 100 µm and 40 µm cell strainers (Falcon). Using RBC lysis buffer (BD Biosciences, Franklin Lakes, NJ, USA) to lyse blood cells, then washed the cell pellet and performed cell counting. For yellow and red bone marrow, tissue pieces were flushed instead of undergoing enzymatic digestion. PBMCs were isolated using a pig peripheral blood lymphocyte separator kit (Solarbio, China). The brain and pancreas were processed for single-cell dissociation according to the GEXSCOPE tissue dissociation kit protocol (Singleron Biotechnologies). All tissue digestion procedures were completed within 16 hours.

#### 2.2.3 Construction and sequencing of single-cell libraries

Single-cell libraries were constructed using GEXSCOPE^®^ Single Cell RNA Library Kits. Briefly, cells were captured and barcoded, followed by reverse transcription of the mRNA captured by the barcoding beads and PCR amplification, and subsequent library preparation. The resulting libraries were sequenced on an Illumina NovaSeq 6000 platform (Illumina Inc., San Diego, CA, USA) to generate high-quality transcriptomic data.

#### 2.2.4 Processing, quality control and integration of scRNA-seq data

Single-cell RNA raw sequencing data were aligned to the Sscrofa 11.1 reference genome using CeleScope v1.1.8, generating raw cell-gene count matrices based on the Sus scrofa GTF (v101) file from Ensembl. Gene symbols for Ensembl IDs without known symbols were assigned by blasting the corresponding protein sequences against the UniProt database. In Scanpy (45), quality control was performed on each sample to filter out low-quality cells [maximal unique molecular identifiers (UMIs) = 30000, maximal number of genes = 5000 (potential doublets), minimum number of genes = 300 or 500, Scrublet (46) doublet detection score < 0.25] (**Supplementary Table 2**). Subsequently, we performed a two-step integration and annotation of all tissues. The first step involved the organ-specific annotation. In brief, the scRNA-seq data from samples of the same organ or physiologically similar organs were merged and performed standard scRNA-seq analysis workflow, including normalization, batch effects correction, dimension reduction, clustering (using the Louvain algorithm), and cell type annotation. Batch effects within organs were corrected using either the harmony (47) or BBKNN procedures (48) in Scanpy v1.7.1 (45), or the FindIntegrationAnchors function in Seurat v4.1.1 (49). Cell type identification was comprehensively defined through automated annotation using CelliD combined with manual validation based on the expression of cell type marker genes and cluster-specific differentially expressed genes (DEGs). The cell type characteristic genes used for CelliD were retrieved from PanglaoDB (https://panglaodb.se/), the Human Protein Atlas (https://www.proteinatlas.org/), and relevant publications. The second step involved the global integration and re-annotation across all organs. First, we integrated the annotated single-cell datasets of all organs. We used Scanorama (50) to correct sample effect, and BBKNN (batch_key = “Platform”, use_rep = “X_scanorama”) to correct platform effects. To cluster single cells by their expression profiles, we applied the Louvain approach at resolution = 3. The final annotation was based on canonical markers, top 200 DEGs, and organ-specific cell type references.

### 2.3 ScRNA-seq data analysis of macrophages

#### 2.3.1 Macrophage data extraction

We extracted macrophages from the single-cell transcriptome atlas of a pregnant sow’s multiple tissues and the male reproductive system of a mated boar (constructed in the above method) following the procedure outlined below and performed single-cell data analysis. First, all immune cells (249,689 cells) were extracted from the atlas and extensively annotated by automatic and manual methods (for detailed descriptions, see Cell type identification). We identified 15 high-quality immune cell types with a resolution of 1 (**Supplementary Figures 1A, C**), which were divided into two major categories: myeloid and lymphoid lineages. Then, a total of 164,113 myeloid cells (Promyelocytes, Erythrocytes, Neutrophils, Monocytes, Plasmacytoid dendritic cells, Dendritic cells, Microglia, and Macrophages) were extracted from the re-annotated immune cell atlas and further annotated, resulting in 39 cell types with a resolution of 4.5 (**Supplementary Figures 1B, D**). In the above process, we excluded potential contaminating cell types based on the expression of top 200 DEGs and non-immune cell canonical marker genes to accurately identify and define the final macrophage population. Finally, we extracted macrophages from the myeloid cell atlas for subsequent analysis.

#### 2.3.2 Dimension reduction and unsupervised clustering

Single-cell RNA-seq data were processed for dimension reduction and unsupervised clustering by following the workflow in Scanpy (45). In brief, 2000 highly-variable genes were selected for downstream analysis by using “scanpy.pp.highly_variable_genes” function with parameter ‘‘n_top_genes = 2000’’. Then, effects on the total counts per cell and the percentage of mitochondrial gene counts were regressed out by using “scanpy.pp.regress_out” function. A principal component analysis (PCA) matrix with 50 components were calculated to reveal the main axes of variation and denoise the data by using “scanpy.tl.pca” function with parameter ‘‘svd_solver = ‘arpack’, n_comps = 50’’. We employed the harmony algorithm to correct batch effects in samples collected from pregnant sows and boars. For visualization, the dimensionality of dataset was further reduced using Uniform Manifold Approximation and Projection (UMAP) implemented in “scanpy.tl.umap” function with default parameters. To cluster single cells by their expression profiles, we used an unsupervised graph-based clustering algorithm called Leiden (resolution=2). The DEGs were identified by using the “scanpy.tl.rank_genes_groups” function with parameter “method = ‘wilcoxon’”.

#### 2.3.3 Cell type identification

We used canonical marker genes collected from the literature and top DEGs combined with the immune cell automatic annotation software CellTypist (51) for cell type annotation. The Python package CellTypist (v.1.5.1) was used to perform annotation prediction with logistic regression models and parameter “majority_voting = True”. Partly clusters sharing >100 of the top 200 DEGs and with similar tissue origins were selectively merged. Clusters mainly (>90%) derived from a specific tissue with an established nomenclature were directly assigned that identity (e.g., Kupffer cells), while clusters lacking a known name or composed of multiple tissues were named by selected representative DEGs.

### 2.4 hdWGCNA analysis

**Figure 1:**
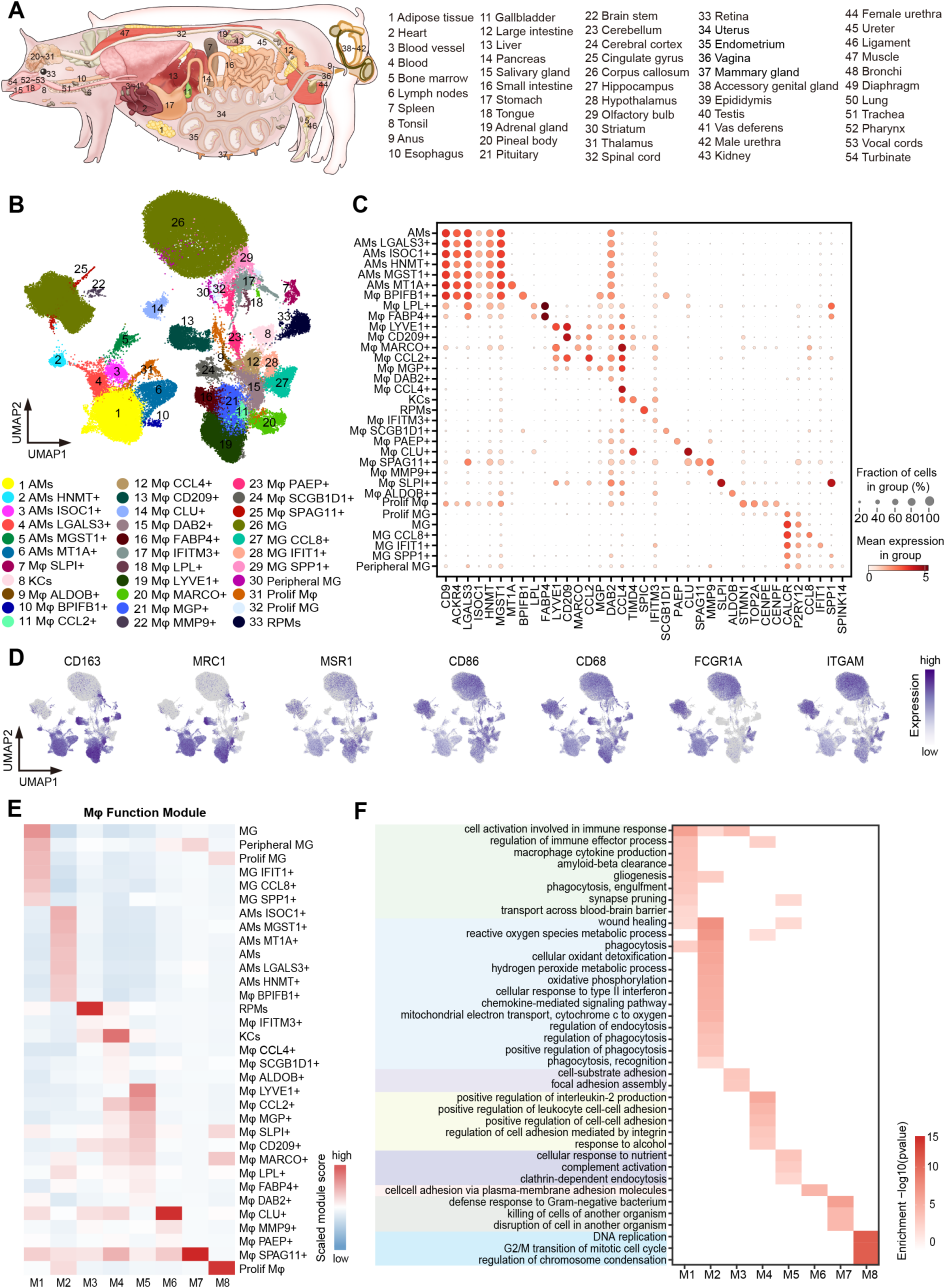
Cross-tissue single-cell transcriptome atlas of pig macrophages. **(A)** Schematic diagram of the anatomy of the 54 tissues/organs involved in this paper. **(B)** UMAP visualization of macrophages (114,881 cells) colored by 33 macrophage subtypes. **(C)** Dot plot showing normalized expression of selected marker genes for macrophage subtypes. The color represents mean expression level, and the size indicates the proportions of cells expressing the genes. **(D)** UMAP visualization of the expression of *CD163*, *MARC1*, *MSR1*, *CD86*, *CD68*, *FCGR1A*, and *ITGAM* in the macrophages. **(E)** The heatmap of the gene module scores of 33 macrophage subtypes. Color scale: red, high score; blue, low score. **(F)** Heatmap showing GO functional enrichment (p < 0.05) of the macrophage gene modules.

Genes were clustered into functional modules using the R package hdWGCNA (0.2.24) (52). Genes that are expressed in at least 0.1% of cells in our dataset were subjected to analysis. Standard parameters were changed to a soft threshold at power of 5 (based on scale free topology model fit, R2 = 0.80), a “signed” network, and a minimum module size of 50. The algorithm assigned 13188 genes to 8 modules as shown in **Figure 1E**.

### 2.5 Gene ontology (GO) enrichment analysis

GO analysis was used in the clusterProfiler v4.10.0 package. The GO terms of selected genes were enriched in the database “org.Hs.eg.db” using “enrichGO” function because of the lack of study in pigs. Benjamini-Hochberg (BH) method was used for the multiple test adjustment.

### 2.6 Gene set scoring analysis

The gene set activity score of the M1 and M2 macrophage polarization-related, PRRs signaling pathways and scavenger receptors (**Supplementary Table 3**) was obtained by using Scanpy’s “scanpy.tl.score_genes” function with parameter ‘‘ctrl_size = 50, n_bins = 25’’ and defined the gene set activity by using the overall expression level of the gene set in each cell type.

### 2.7 Immunofluorescence staining and imaging

Paraffin-embedded lung tissue was sectioned in a thickness of 3-5 μm using a microtome and adhered to glass slides. Tissue sections underwent deparaffinization, rehydration, and epitope retrieval. Endogenous peroxidase activity was blocked with 3% Bovine Serum Albumin for 30 min. The tissue sections were incubated with primary anti-CD68 mouse antibody (Servicebio; GB123150; 1:200 dilution) and anti-PLSCR1 rabbit antibody (Servicebio; GB113827; 1:500 dilution) overnight at 4 °C. After washing, tissue sections were incubated with fluorescence-conjugated secondary antibodies [Cy3 conjugated goat anti-mouse IgG (Servicebio; GB21301; 1:300 dilution) and Alexa Fluor 488 conjugated goat anti-rabbit IgG (Servicebio; GB25303; 1:400 dilution)] for 50 min at room temperature. Nuclei were stained with DAPI, followed by mounting. To obtain multispectral images, the stained slide was scanned using the Pannoramic MIDI, and the images were viewed by CaseViewer software.

### 2.8 Integration of uterine macrophage datasets from pregnant and non-pregnant sows

Firstly, we merged the filtered gene expression matrices of the uterine horn macrophage datasets from pregnant and non-pregnant individuals. Subsequently, batch correction was performed with harmony. Unsupervised Leiden clustering was further performed with a resolution of 1.5, and visualization was done using UMAP on the corrected combined data. Finally, “FindAllMarkers” function implemented in Seurat v4 (49) was used to identify DEGs across clusters with the options “logfc.threshold = 1”. Multiple test correction for P value was performed using the Bonferroni method, and 0.05 was set as a threshold to define significance. We used volcano plot to visualize DEGs based on gene expression after the log-transformed.

### 2.9 Cross-species comparison of macrophages between pig, human, and mouse

We downloaded single-cell transcriptome dataset of human decidua during pregnancy (53), and extracted annotated macrophages for subcluster analysis, resulting in 4, 857 cells classified into 10 subtypes. MetaNeighbor (54) was used to assess the similarity between human and porcine macrophage subtypes (this study).

We integrated single-cell data from 11 shared organs (adrenal gland, blood, bone marrow, kidney, liver, muscle, pancreas, small intestine, spleen, stomach, and uterus) of the pigs (this study), humans (55), and mice (56) using Scanpy (45). We annotated and extracted macrophages for subsequent analysis. Harmony procedure (47) with the parameter “key = ‘species’” was applied to generate corrected PCA coordinates. We used the BBKNN procedure (48) with the parameters “batch_key = ‘tissue’, use_rep = ‘X_pca_harmony’” to further integrate the tissues. Cell clustering was performed using the Leiden algorithm with resolution = 1. We used MetaNeighbor (54, 57) to assess the similarity between macrophage subtypes among the species.

### 2.10 Statistical analysis

Statistical analyses were done using R software. Unless otherwise stated in the figure legends, the statistical test for single-cell data analysis was Wilcoxon rank-sum test. The correspondence between symbols and significance values: * p < 0.05; ** p < 0.01; *** p < 0.001; **** p < 0.0001.

## 3 Results

### 3.1 Cross-tissue single-cell transcriptome atlas of pig macrophages

To construct a comprehensive single-cell transcriptome atlas of pig macrophages during pregnancy, we analyzed macrophages from the single-cell transcriptome atlas of individual pregnant sow’s 49 tissues and the male reproductive system of a mated boar constructed in our laboratory (Materials and methods; **Figure 1A**). We conducted detailed cell type annotation, starting from immune cells and progressing to myeloid cells and macrophages, based on the automated immune cell annotation software CellTypist (51) combined with canonical marker genes (**Figure 1B**; **Supplementary Figure 1**). In our analysis, we removed tissues with fewer than 50 cells, and ultimately obtained a total of 114,881 macrophages across 54 tissues/organs, with an average number of genes ranging from 457 in the retina to 2,299 in the epididymis (**Supplementary Table 1**). By integrating all macrophages and systematically performing scRNA-seq analysis on the integrated dataset, we grouped macrophages into 33 subtypes based on the top 200 DEGs, and they co-expressed a combination of macrophage markers, such as *CD163*, *MRC1*, *FCGR1A*, and *ITGAM* (**Figures 1B–D**; **Supplementary Table 4**). Well-known resident macrophage subtypes were enriched in corresponding tissues, for example, microglia (MG) in the CNS, Kupffer cells (KCs) in the liver, red pulp macrophages (RPMs) in the spleen, and alveolar macrophages (AMs) in the lung (6, 58–60).

Activated macrophages usually possess binary polarization, including M1 macrophages and M2 macrophages, which are mainly involved in pro-inflammatory responses and anti-inflammatory responses, respectively (61, 62). To understand the polarization states of macrophage subtypes, we compiled M1 and M2 macrophage-related gene sets from the literature and calculated the corresponding gene set scores as the M1 and M2 scores (**Supplementary Table 3**). Most of the 33 macrophage subtypes were biased toward M2 polarization (**Supplementary Figure 2A**), and they all highly expressed M2 marker genes such as *CD163* and *MRC1* (**Figure 1D**).

We utilized hdWGCNA (52) for gene co-expression network analysis, aiming to uncover key gene modules and their enrichment in different macrophage subtypes. We identified eight key co-regulated gene modules (M1~M8) (**Figure 1E**; **Supplementary Figure 2B**) and performed GO enrichment analysis on the core genes of each module to investigate their function features (**Figure 1F**). The microglia-related M1 module was enriched in genes associated with “gliogenesis”, “synapse pruning”, and “transport across the blood-brain barrier”. In contrast, the lung macrophage-related M2 module was linked to the metabolic pathways, including “reactive oxygen species metabolic process” and “hydrogen peroxide metabolic process”, as well as other pathways such as “cellular oxidant detoxification”, “phagocytosis”, and “cellular responses to type II interferon”. Additionally, the M7 module was specifically enriched in Mφ SPAG11+ confined to the epididymis, which was involved in the “defense response to Gram-negative bacterium” and “disruption of cell in another organism” (**Figure 1F**).

### 3.2 Revealing tissue-specific and shared macrophage subtypes

**Figure 2:**
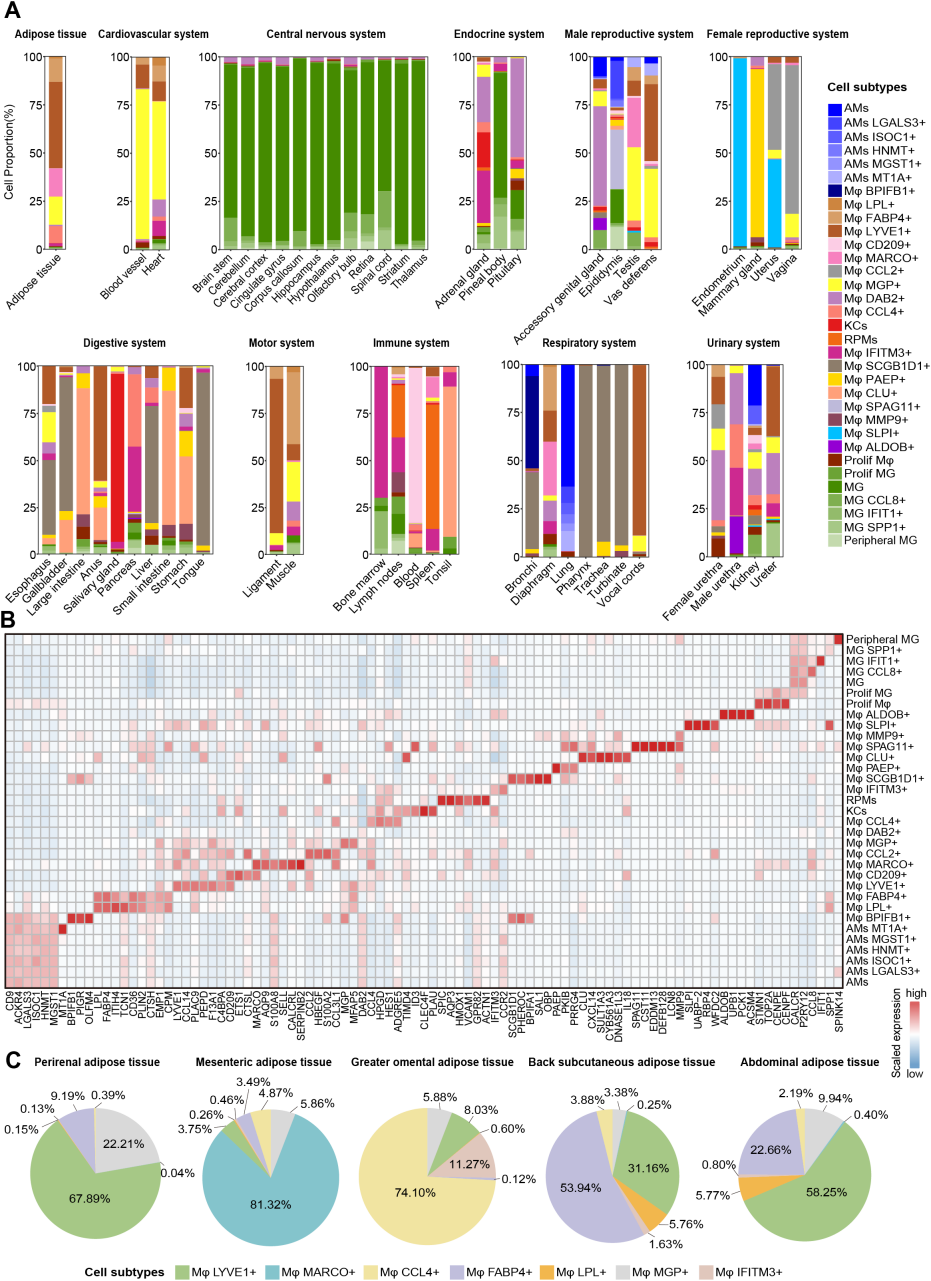
Heterogeneity and spatial dynamics of macrophages. **(A)** Bar plots showing the macrophage subtypes compositions at each tissue/organ in different systems. **(B)** Heatmap depicting the expression of manually selected top DEGs in 33 macrophage subtypes. Color scale: red, high expression; blue, low expression. **(C)** Pie chart showing the proportion of Mφ LYVE1+, Mφ MARCO+, Mφ CCL4+, Mφ FABP4+, Mφ LPL+, Mφ MGP+, and Mφ IFITM3+ in different adipose tissue after removing other macrophage subtypes.

Extensive tissue coverage is advantageous in elucidating the tissue-specific and shared characteristics of macrophage subtypes among tissues. Recent studies on human prenatal immune cell development across tissues have identified microglia in peripheral tissue, such as fetal skin, testis, and heart (42). In our study, we classified microglia into six distinct subtypes [MG, MG IFIT1+, MG CCL8+, MG SPP1+, peripheral microglia (Peripheral MG), and Proliferating microglia (Prolif MG)], all of which exhibited high expression of the marker genes *CALCR*, *C3* and *P2RY12* (**Figures 1B, C**). Microglia represented the dominant cell type in the CNS, but MG subtypes were also detected in other tissues, such as Peripheral MG existed in the epididymis, consistent with findings in humans (42, 63), suggesting conservation of this cell type across species. In addition, MG IFIT1+ cells were detected in the blood, whereas other MG subtypes displayed heterogeneous distributions across the male reproductive and urinary systems. Interestingly, we also observed similar phenomena in other tissue-resident macrophages. For instance, KCs were found in the adrenal gland, RPMs were present in the bone marrow, and AMs were identified in the male urethra (**Figure 2A**; **Supplementary Figure 2C**).

In addition, we identified several tissue- or system-specific macrophage subtypes. Mφ PAEP+ (*PKIB* and *PRRG4*), Mφ SPAG11+ (*CST11*, *EDDM13*, *DEFB128*, and *LCN8*), Mφ BPIFB1+ (*PIGR* and *OLFM4*), Mφ CD209+ (*ETS1* and *CTSL*), and Mφ ALDOB+ (*UPB1*, *PCK1*, and *ACSM4*) were found to be restricted to specific tissues, including the mammary glands, epididymis, bronchi, lymph nodes, and kidneys, respectively (**Figures 2A, B**; **Supplementary Figure 2C**; **Supplementary Table 4**). We also observed two female reproductive system-specific macrophage subtypes: Mφ CCL2+ expressed high levels of *HBEGF*, *S100A2*, and *CCL3L1* in the uterus and vagina, and Mφ SLPI+ highly expressed *UABP-2*, *RBP4*, and *WFDC2* in the uterus and endometrium (**Figures 2A, B**; **Supplementary Figure 2C**; **Supplementary Table 4**). Furthermore, we also identified tissue-shared macrophage subtypes in our atlas. For example, Mφ DAB2+ subtype was the most widely conserved macrophage subtype across tissues, identified in 45 tissues. Mφ SCGB1D1+ subtype (*PHEROC*, *BPIFA1*, *SAL1*, and *OBP*) was found in the upper respiratory and digestive tract tissues, including the tongue, pharynx, trachea, esophagus, and turbinate (**Figures 2A, B**; **Supplementary Figure 2C**).

Notably, seven distinct macrophage subtypes were principally identified across adipose tissue, with each fat depot (perirenal adipose tissue, mesenteric adipose tissue, greater omental adipose tissue, back subcutaneous adipose tissue, and abdominal adipose tissue) harboring a subset of these subtypes, reflecting significant heterogeneity based on anatomical location (**Figure 2C**). The Mφ LYVE1+ and the Mφ MGP+ were present in various proportions across all fat depots, and the former was one of the predominant subtypes in perirenal, back subcutaneous, and abdominal adipose tissue. Mφ FABP4+ and Mφ LPL+ shared high expression of *CD36*, *FABP5*, *ITIH4*, and *PLIN2*, with lower or almost absent proportions in mesenteric, perirenal, and greater omental adipose tissue compared to the other two adipose tissues. Mesenteric adipose tissue uniquely harbored the Mφ MARCO+ subtype with high expression of *AQP9*, *S100A8*, *SELL*, and *CALCRL*; greater omental adipose tissue was predominantly composed of Mφ CCL4+ and Mφ IFITM3+ with high expression of *HPGD* and *HES1* (**Figure 2B**; **Supplementary Table 4**). These findings highlighted the heterogeneity of macrophage composition between different anatomical sites of the same tissue. In brief, we generated a cross-tissue macrophage atlas of a pregnant pig and preliminarily explored the distribution characteristics of macrophages between tissues, laying the foundation for studying their functional heterogeneity.

### 3.3 Heterogeneity of immune and phagocytic functions among macrophage subtypes

**Figure 3:**
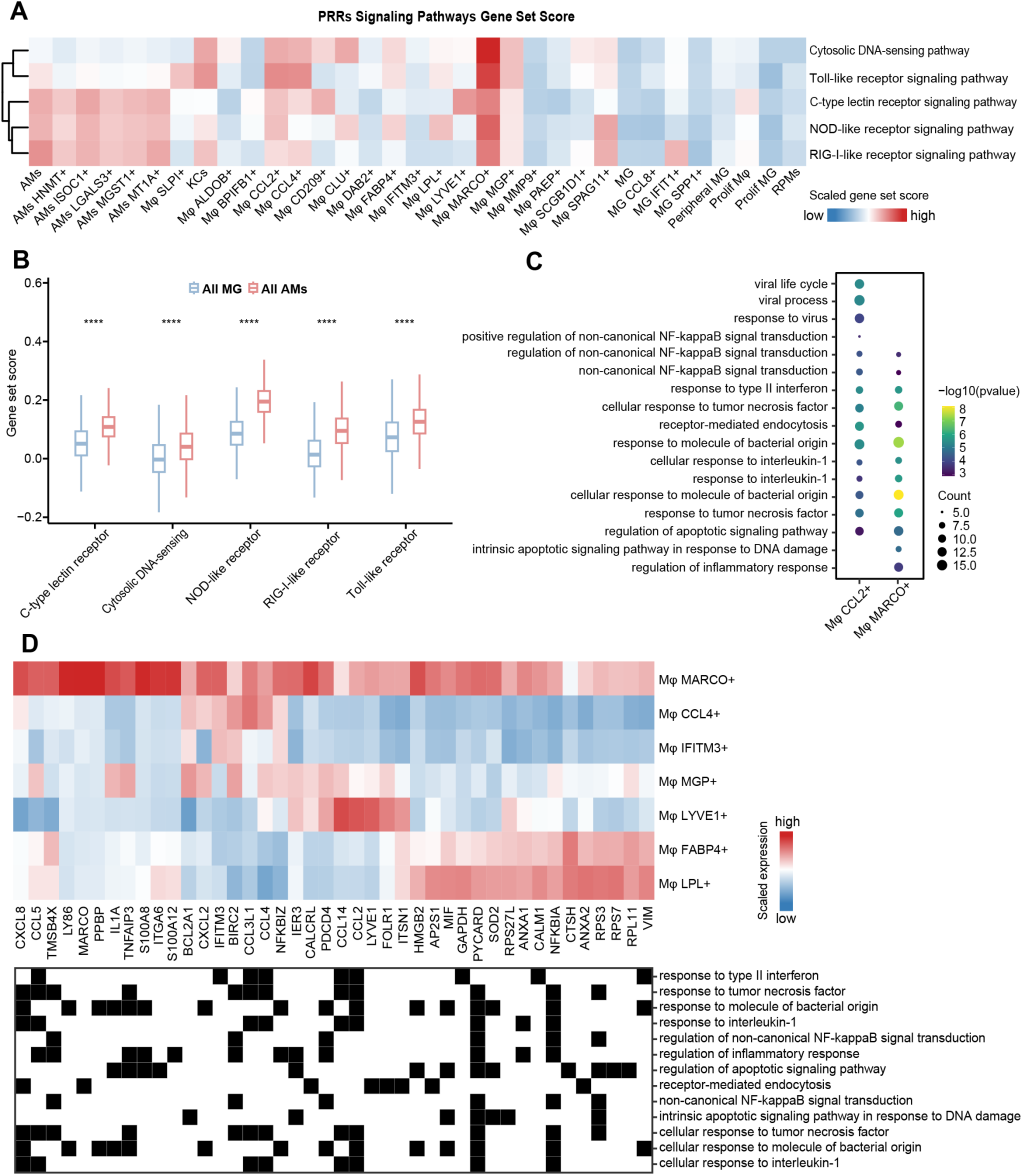
Heterogeneity of immune function among macrophage subtypes. **(A)** Heatmap showing the gene set scores of PRRs signaling pathways. Color scale: red, high score; blue, low score. **(B)** Comparison of gene set scores for PRRs signaling pathways between all MG subtypes and all AMs subtypes. The significance levels are marked by asterisks (Wilcoxon rank sum test, ****p < 0.0001 **(C)** Bubble plot visualizing GO enrichment analysis (p < 0.05) of DEGs in Mφ CCL2+ and Mφ MARCO+. The dot size represents the number of genes involved in the relevant term. The color bar indicates the enrichment significance. **(D)** The expression of genes related to functional terms enriched by Mφ MARCO+ in **(C)** in seven types of adipose tissue-associated macrophages (top). Color scale: red, high expression; blue, low expression. The functional terms to which the genes belong are shown (bottom).

Macrophages express various pattern recognition receptors (PRRs) that can recognize the specific molecular structure on the surface of pathogens, apoptotic and damaged cells, performing functions like phagocytosis, clearance, killing, and antigen presentation, etc. (64–66). We provided a rich resource to investigate the differences in PRRs functional activity among macrophage subtypes, which may reveal the influence of tissue microenvironment on macrophage functional plasticity. We downloaded genes related to five major PRRs signaling pathways from the KEGG database, including Toll-like receptors, RIG-I-like receptors, NOD-like receptors, C-type lectin-like receptors, and Cytosolic DNA sensors, and then performed gene set scoring for each pathway (**Figure 3A**). Our results showed that the activity level of the PRRs signaling pathways varied among different macrophage subtypes, and their characteristics may be related to the physiological properties of tissues. AMs subtypes showed high scores for PRRs signaling pathways, including RIG-I-like receptors, NOD-like receptors, and C-type lectin-like receptors (**Figures 3A, B**), likely due to their frequent response to various pathogen exposures in the lungs. Conversely, all the PRRs signaling pathways scores in all MG subtypes were relatively low compared to other subtypes (**Figures 3A, B**), which could be attributed to the protective role of the blood-brain barrier in reducing the susceptibility of brain tissue to pathogen infection. This difference is closely related to the physiological properties and the degree of pathogen exposure of the two tissues, reflecting their adaptability in immune defense strategies. Both Mφ MARCO+ and Mφ CCL2+ had high gene set scores in the five types of PRRs signaling pathways, and their DEGs were significantly enriched in other synergistic pathways, such as NF-kappaB signal-related functions (67), interferon response, and killing of bacteria and viruses (**Figure 3C**). Interestingly, compared with Mφ MARCO+ in mesenteric adipose tissue, Mφ CCL4+, Mφ IFITM3+, Mφ MGP+, Mφ LYVE1+, Mφ FABP4+, and Mφ LPL+ subtypes predominantly found in other fat depots showed relatively low activity in PRRs signaling pathways. To further investigate the heterogeneity of PRRs signaling activity among the seven macrophage subtypes, we excluded cells from other tissues and only compared their gene expression patterns in adipose tissue (**Figures 2C**, **3D**). The results showed that Mφ MARCO+ highly expressed CXCL (*CXCL8* and *CXCL2*) and CCL (*CCL2*, *CCL4*, *CCL5*, and *CCL14*) family genes, which play an important role in the recruitment and migration of immune cells. Previous studies have shown that mesenteric adipose tissue can be regarded as a second barrier similar to the epithelial barrier, which can prevent the systemic transmission of potentially harmful bacteria through the intestinal cavity in the host, and is strongly regulated by the innate immune system (68–70).

**Figure 4:**
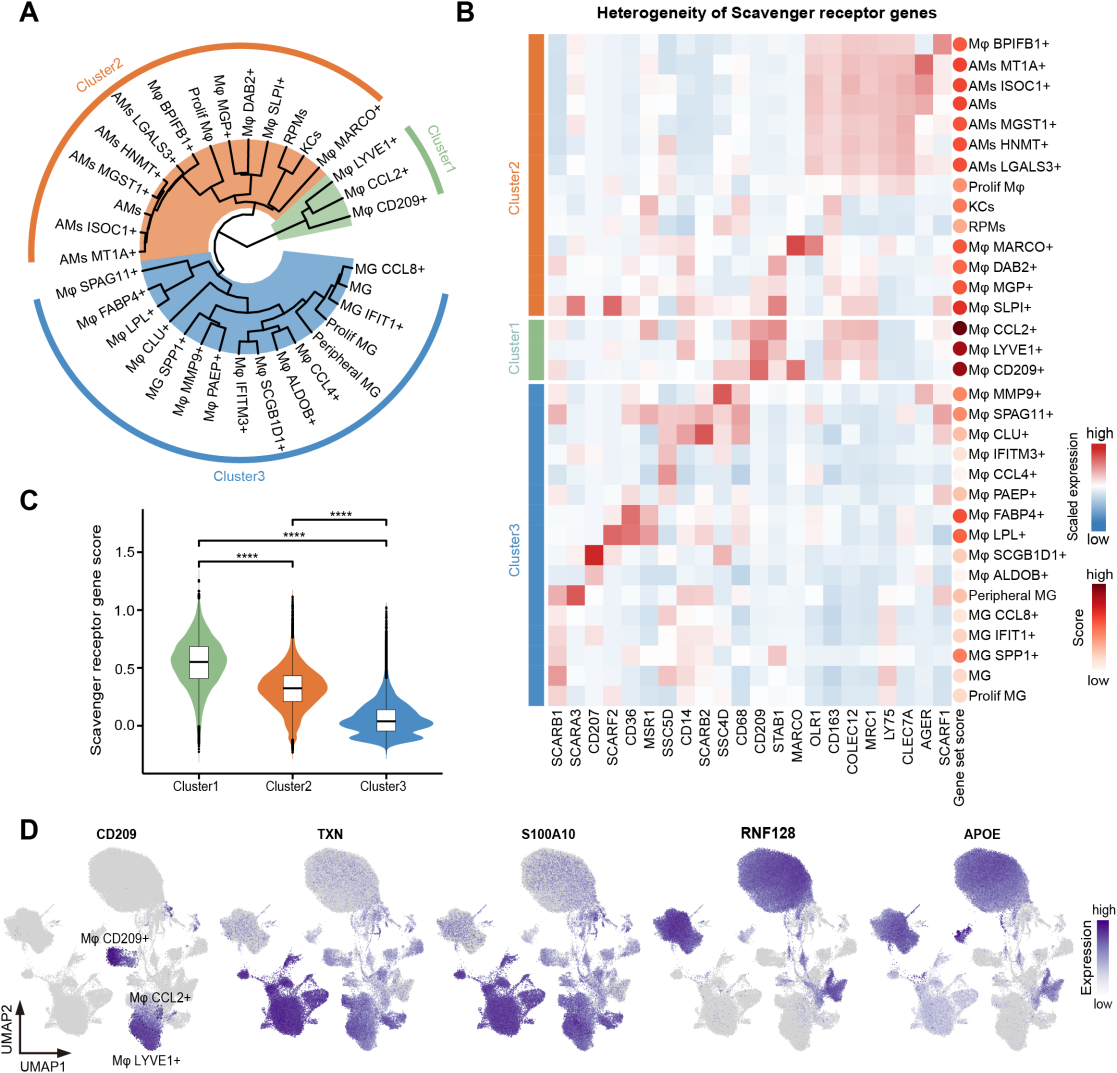
Heterogeneity of phagocytic function among macrophage subtypes. **(A)** Unsupervised hierarchical clustering of the 33 macrophage subtypes. Three clusters were dissected and marked as green (cluster 1), orange (cluster 2), and blue (cluster 3). **(B)** Heatmap showing the expression of scavenger receptor genes in 33 macrophage subtypes. Cell types were arranged according to the classification in **(A)**. Color scale: red, high expression; blue, low expression. The dots on the right represent the gene set score of scavenger receptor genes. The redder the dot, the higher the score. **(C)** Violin plot showing the scavenger receptor gene set score of three clusters. Interquartile ranges (IQRs) as boxes, with the median as a black line and the whiskers extending up to the most extreme points within 1.5-fold IQR, the outliers are shown as individual points. The significance levels among cluster 1, cluster 2, and cluster 3 are marked by asterisks (Kruskal-Wallis test, ****p < 0.0001). **(D)** UMAP visualization of the expression of *CD209*, *TXN*, S*100A10*, *RNF128*, and *APOE* in the macrophages.

Scavenger receptors facilitate the phagocytosis of various substances by binding to specific ligands and play a crucial role in maintaining tissue homeostasis, immune surveillance, and pathogen clearance (71). We next explored the expression pattern of 22 scavenger receptors (72) obtained from the HUGO Gene Nomenclature Committee (HGNC) across all macrophage subtypes. An unsupervised hierarchical clustering analysis of the transcriptome patterns yielded three clusters, with the activity score of the scavenger receptor gene set decreasing progressively from cluster 1 to cluster 3 (**Figures 4A, C**). These clusters were characterized by differential expression of *CD209*, *TXN*, *S100A10*, *RNF128*, and *APOE* (**Figure 4D**); however, the expression patterns of scavenger receptor genes lacked a regular pattern among clusters. Cluster 1 consisted of three distinct macrophage subtypes: Mφ CCL2+, Mφ CD209+, and Mφ LYVE1+, predominantly enriched in the female reproductive system, lymph nodes, and adipose tissue, respectively. These subtypes displayed high expression of key scavenger receptor genes, including *CD68*, *CD209*, *MRC1*, *CD163*, and *STAB1* (**Figure 4B**). Cluster 2 primarily included macrophage subtypes present in the lung (AMs), liver (KCs), spleen (RPMs), endometrium and uterine horn (Mφ SLPI+), and mesenteric adipose tissue (Mφ MARCO+), etc. All AM subtypes exhibited high expression of *COLEC12*, *CLEC7A*, and *LY75* (**Figure 4B**). Notably, we observed that most scavenger receptor genes were expressed in Mφ SLPI+. Together, female reproductive system-specific Mφ CCL2+ and Mφ SLPI+ had very high scavenger phagocytic activity. We speculated that this is related to special physiological changes in the female reproductive system during pregnancy. All MGs subtypes were classified into cluster 3, showing high expression of *SCARB1*, as well as different expression patterns of *SCARA3*, *CD207*, *CD36*, and *SSCD5D* (**Figure 4B**).

### 3.4 Cross-tissue expression patterns of TFs, cytokines and cell surface receptors

**Figure 5:**
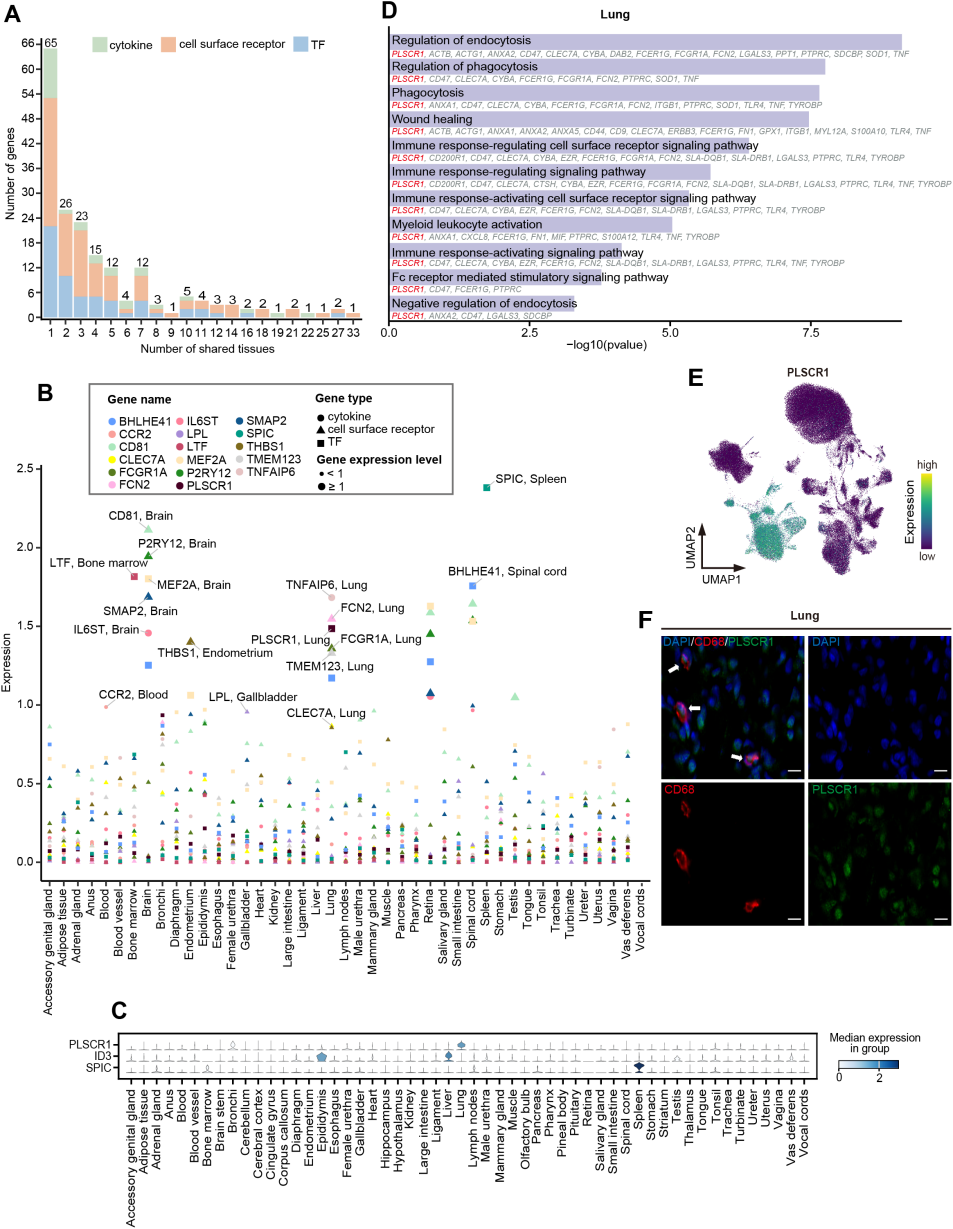
Cross-tissue expression patterns of TFs, cytokines, and cell surface receptors. **(A)** Stacked bar chart showing the number of highly expressed TFs (blue), cytokines (green), and cell surface receptors (orange) expressed in different numbers of tissues. The number above each bar represents the total number of genes. **(B)** Scatter plot showing the expression of tissue-highly expressed genes that are highly expressed only in one tissue. The color of the dots represents different genes, the shape of the dots represents different gene types, and the size of the dots represents the level of gene expression. The larger the dot, the greater the expression. **(C)** Stacked violin plot depicting normalized expression of tissue-specific TFs. The color represents median expression level. **(D)** GO enrichment analysis (p < 0.05) of DEGs in lung macrophages. The genes involved in the GO terms are shown below the corresponding term. **(E)** UMAP visualization of the expression of *PLSCR1* in the macrophages. **(F)** Representative Immunofluorescence staining images of DAPI, CD68, and PLSCR1 in lung tissue. Scale bars, 10 μm.

We are interested in understanding the cross-tissue expression pattern of TFs, cytokines, and cell surface receptors, which are critical for the function and fate specialization of macrophages (73). We downloaded TFs, cytokines, and cell surface receptors from AnimalTFDB, HPA (The Human Protein Atlas), and UniProtKB. Then, we overlapped them with the genes in our dataset, retaining 1,215 TFs, 154 cytokines, and 775 cell surface receptors for subsequent analysis (**Supplementary Table 5**). Tissue highly expressed genes were defined as those expressed in over 50% of cells within a given tissue, with an average expression level at least 1-fold higher than in other tissues (p<0.05, pts>0.5, log2FC>1). Our analysis identified a total of 59 TFs, 28 cytokines, and 101 cell surface receptors covering 43 tissues, most of which exhibited obvious tissue-specific high expression profiles (**Figures 5A, B**; **Supplementary Figures 3, 4**; **Supplementary Table 6**).

A panel of well-known lineage-determining and function regulation TFs were identified in specific tissues, such as *SPIC* for splenic red pulp macrophages (14), *LTF* for bone marrow macrophages (74), and *MEF2A* for microglia of the brain (75, 76). We observed that *ID3*, critical for the specification and development of Kupffer cells (11), was highly expressed not only in the liver but also in the epididymis (**Figure 5C**). Moreover, we found that lung macrophages specifically expressed high levels of *PLSCR1*, a key regulator in the innate type 2 immune response and antiviral response in the lungs (77, 78) (**Figures 5C, E**). Immunofluorescence staining revealed the expression of PLSCR1 in lung macrophages (**Figure 5F**). GO enrichment analysis of the DEGs of lung macrophages indicated that *PLSCR1* may be involved in immune and phagocytosis-related functions together with *ANXA1*, *ANXA2*, and *FCGR1A* (**Figure 5D**). We noticed that *FOS* was highly expressed in the widest range of tissues (n=27) and was enriched in pathways related to bacterial and viral resistance across tissues (**Supplementary Figure 3A**; **Supplementary Tables 6, 7**), suggesting that *FOS* may be one of the central TFs shared by macrophages in immune regulation among tissues.

The 28 identified cytokines primarily included the CCL family, CXCL family, interleukins (IL) family-related genes, and tumor necrosis factors (TNF) family-related genes (**Supplementary Figure 3B**; **Supplementary Table 6**). Among them, the members of TNF family-related genes exhibited strong tissue-specific expression pattern, such as *TNF* and *TNFAIP6* in the lung, *TNFSF12* and *TNFSF13B* in the epididymis, *TNFAIP8* in the testis, as well as *TNFRSF1B* in the accessory genital gland. Additionally, *IL6ST*, which is essential for astrocyte differentiation and neuronal survival (79, 80), was specifically highly expressed in the brain, while *IL1A* and *ILF3* were highly expressed in the testis (**Figure 5B**; **Supplementary Figure 4**; **Supplementary Table 6**). Conversely, the high expression patterns of CCL family genes (*CCL2*, *CCL3L1*, *CCL4*, *CCL5*, *CCL8*, and *CCL14*) and CXCL family genes (*CXCL8*, *CXCL2*, and *CXCL14*) were observed in at least four tissues, suggesting that the recruitment and migration signals released by macrophages exhibit certain commonalities across different tissues.

The 101 cell surface receptors ranged from 2 in the female urethra (retina and spinal cord) to 48 in the epididymis. The tissue-specific cell surface receptors were predominantly identified in the lung (*CCR1*, *CD200R1*, *CD48*, *CLEC7A*, *FCGR1A*, *FCN2*, *PLSCR1*, *TGFBR1*, *TMEM123*, and *TNF*), epididymis (*ADAM9*, *ANO6*, *CADM1*, *CLEC5A*, *GGA2*, *LRPAP1*, *MERTK*, *MILR1*, and *VAV1*), and brain (*P2RY12*, *CD81*, and *SMAP2*), involving in multiple functions like angiogenesis, cell migration, proliferation and differentiation, endocytosis, and innate immune response. For shared expression patterns across tissues, *ERBB3* (Erb-B2 Receptor Tyrosine Kinase 3), *FOLR1* (Folate Receptor Alpha), *RPSA* (Ribosomal Protein SA), *BIRC2* (Baculoviral IAP Repeat Containing 2), and *TYROBP* (Transmembrane Immune Signaling Adaptor TYROBP) were identified as highly expressed in at least 20 tissues (**Figure 5B**; **Supplementary Figure 3C**; **Supplementary Table 6**).

### 3.5 Changes in the function of uterine macrophages during pregnancy

**Figure 6:**
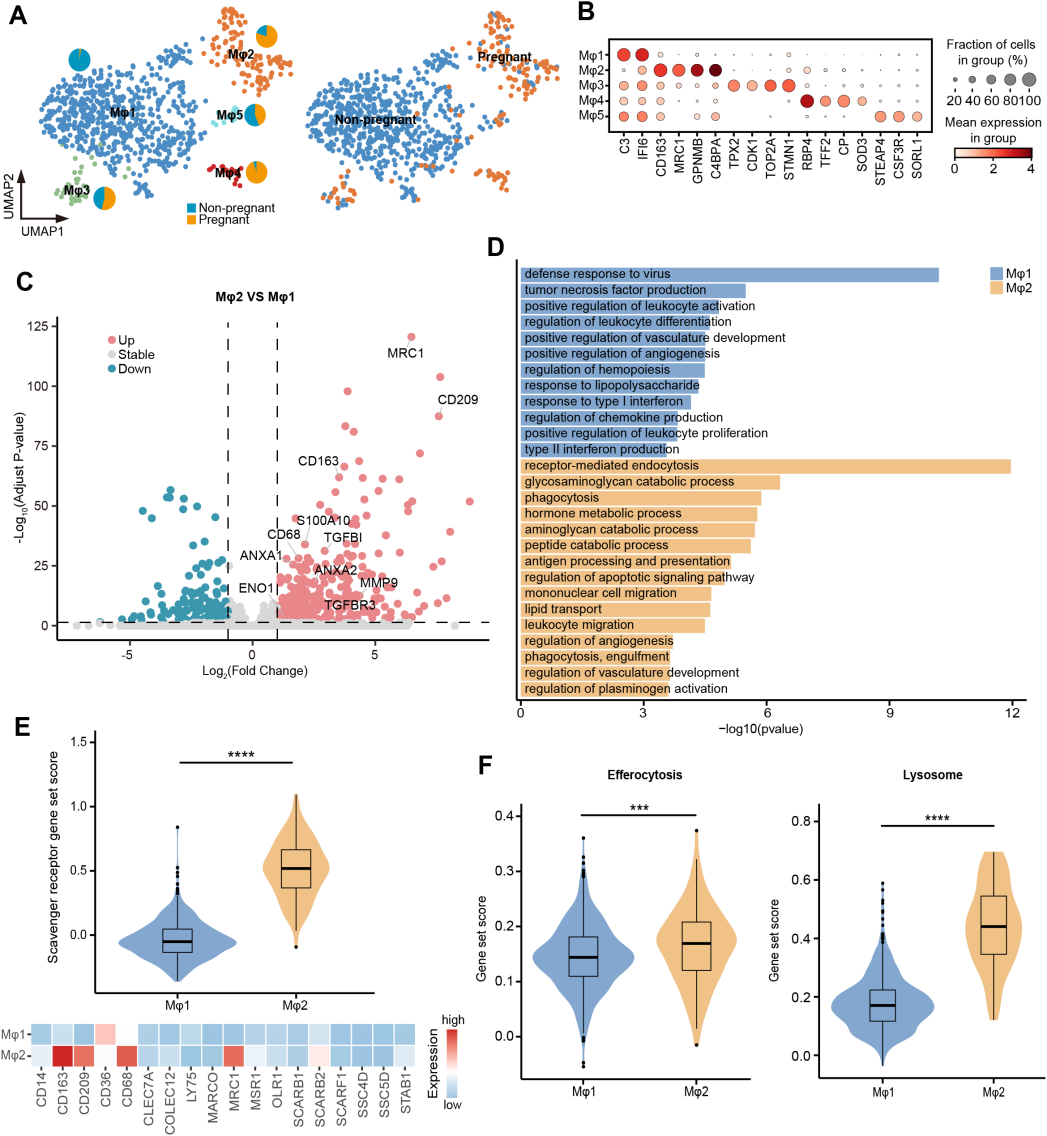
Changes in the function of uterine macrophages during pregnancy. **(A)** UMAP visualization of uterine macrophages colored by cell types (left) and individuals (right). Pie chart showing the proportion of individuals in Mφ1~Mφ5. **(B)** Dot plot displaying normalized expression of selected marker genes for Mφ1~Mφ5. The color represents mean expression level, and the size indicates the proportions of cells expressing the genes. **(C)** Volcano plot showing the DEGs between Mφ1 and Mφ2. Genes with log2 (fold-change) beyond 1 or below -1 with adjusted p value lower than 0.05 were considered as significantly differential expression. Genes that are significantly up-regulated in Mφ2 compared to Mφ1 are shown in red, genes that are significantly down-regulated are shown in blue, and genes with no significant difference are shown in gray. **(D)** GO enrichment analysis (p < 0.05) of DEGs in Mφ1 (blue) and Mφ2 (orange). **(E)** Violin plot (top) showing the scavenger receptor gene set score of Mφ1 and Mφ2. Heatmap (bottom) showing the expression of scavenger receptor genes in Mφ1 and Mφ2. Color scale: red, high expression; blue, low expression. **(F)** Violin plot showing the gene set score of efferocytosis and lysosome. IQRs as boxes, with the median as a black line and the whiskers extending up to the most extreme points within 1.5-fold IQR, the outliers are shown as individual points. Significant difference between Mφ1 and Mφ2 is marked by asterisks (Wilcoxon rank sum test, ***p < 0.001; ****p < 0.0001).

To gain a deeper understanding of the cell state and functional changes of uterine macrophages during pregnancy, we performed single-cell transcriptome sequencing on the uterus of non-pregnant sows, followed by integration with datasets from pregnant sows and extracted macrophages for subcluster analysis (Materials and methods). Our analysis revealed five distinct subtypes (Mφ1~Mφ5) that exhibited unique distribution patterns between pregnant and non-pregnant individuals (**Figure 6A**). Mφ1 predominated in the non-pregnant uterus and highly expressed *SAT1*, *OAS2*, and *IFI6* genes associated with antiviral and pro-inflammatory responses. In contrast, Mφ2 was dominant in the pregnant uterus, characterized by high expression of M2 macrophage markers (*CD209*, *MRC1*, and *CD163*), indicating a shift toward an anti-inflammatory and tissue remodeling phenotype. Mφ3, a subtype characterized by high expression of proliferation-related genes such as *TPX2*, *CDK1*, and *TOP2A*, was present in both pregnant and non-pregnant individuals, although the number of cells was relatively small (**Figures 6A, B**).

To further explore the functional adaptation of macrophages during pregnancy, we compared the gene expression patterns between Mφ1 and Mφ2. The GO enrichment analysis of DEGs revealed that Mφ1 upregulated genes involved in defense response to virus, tumor necrosis factor production, positive regulation of leukocyte activation, and response to type I interferon, while Mφ2 showed significant enrichment in receptor-mediated endocytosis, phagocytosis, and regulation of plasminogen activation (**Figures 6C, D**; **Supplementary Table 8**). Consistently, Mφ2 exhibited significantly higher gene set scores for scavenger receptors, efferocytosis, and lysosome-related pathways compared to Mφ1, supporting its enhanced phagocytic capability (**Figures 6E, F**). In addition, catabolic process were significantly enriched in Mφ2, including glycosaminoglycan catabolic process (*LYVE1*, *CD4*, *GNS*, *GLB1*, *GUSB*, *HEXB*, and *SGSH*), aminoglycan catabolic process (*LYVE1*, *CD4*, *GNS*, *GLB1*, *GUSB*, *HEXB*, and *SGSH*), and peptide catabolic process (*CTSH*, *ECE1*, *CPQ*, *TPP1*, *ACE*, and *NPEPPS*) (**Figure 6D**). This functional enrichment suggested that regulation of the uterine tissue microenvironment and function during late pregnancy was accompanied by active metabolic reprogramming. Taken together, these findings provided a preliminary insight into the dynamic functions of uterine macrophages, highlighting their critical roles in adapting to the physiological demands of late pregnancy.

### 3.6 Conservation and heterogeneity of macrophages across species

**Figure 7:**
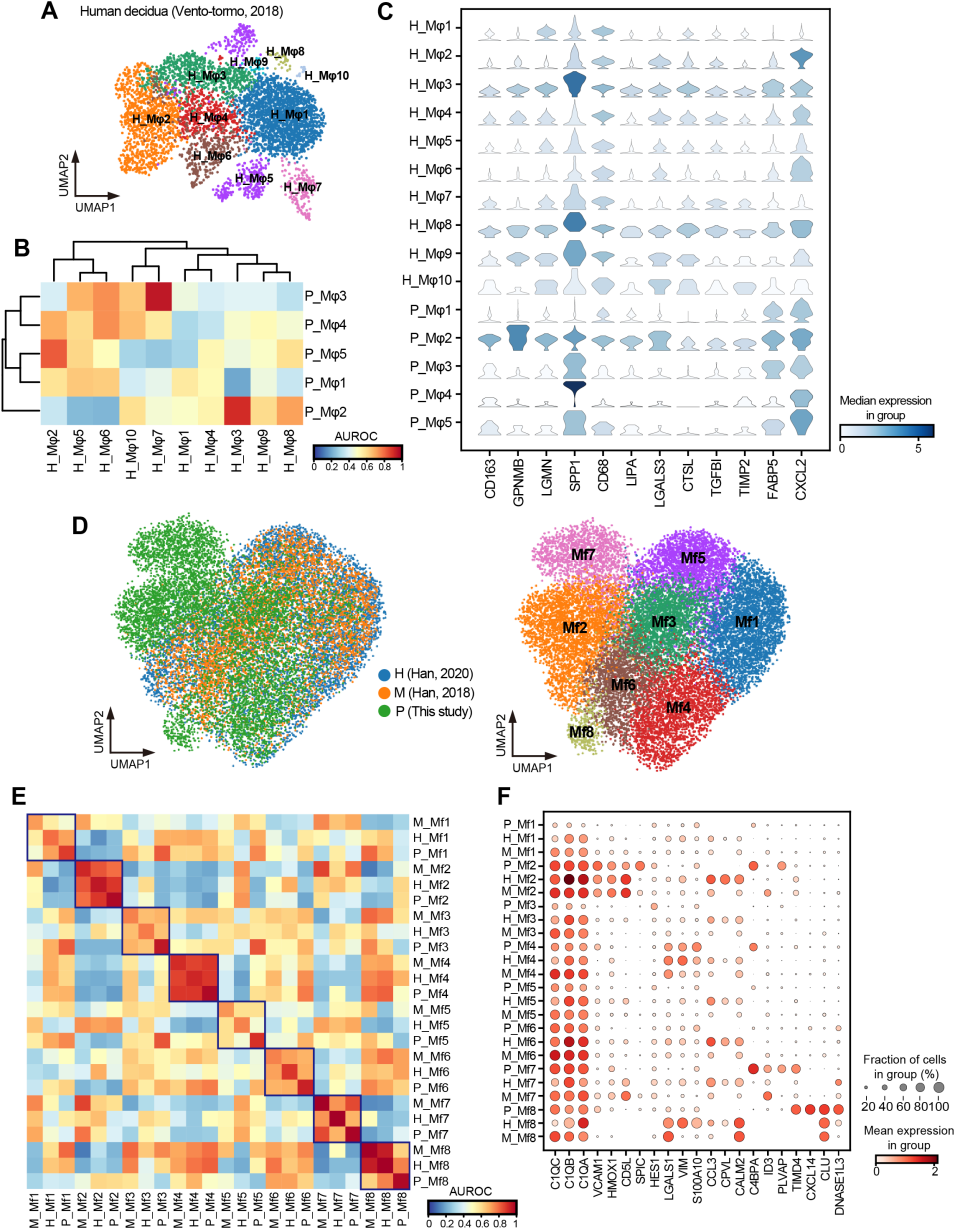
Cross-species comparison of macrophages between pig, human, and mouse. **(A)** UMAP visualization of human decidual macrophages colored by subtypes. **(B)** Heatmap of AUROC scores between macrophage subtypes in humans and pigs based on the highly variable gene set. **(C)** Stacked violin plot showing the shared high expression genes of H_Mφ3, H_Mφ8, and P_Mφ2. **(D)** UMAP visualization of integrated macrophages in humans, pigs, and mice, colored by species (left) and subtypes (right). H: human; M: mouse; P: pig. **(E)** Heatmap of AUROC scores between macrophage subtypes in humans, pigs, and mice based on the highly variable gene set. **(F)** Dot plot showing normalized expression of selected DEGs for macrophage subtypes.

To preliminarily investigate whether the characteristics observed in pregnant sow uterine macrophages are conserved in humans, we downloaded single-cell transcriptome data of human decidua during pregnancy (53), extracted annotated macrophages for subcluster analysis (H_Mφ1~H_Mφ10) (**Figure 7A**). We used MetaNeighbor (54) to correlate the human decidual macrophage subtypes with the pregnant and non-pregnant uterine macrophage subtypes (this study, **Figure 6A**) and found that different subtypes exhibited various degrees of similarity. Among them, H_Mφ3 (AUROC score = 0.91) and H_Mφ8 (AUROC score = 0.71) of humans showed the highest similarity with P_Mφ2, which was mainly derived from pregnant sow uterine macrophages, and showed low similarity with P_Mφ1, which primarily derived from non-pregnant sow uterine macrophages (**Figure 7B**). Notably, H_Mφ3 and H_Mφ8 shared similar molecular expression patterns with P_Mφ2, including high expression of *CD163*, *GPNMB*, *LGMN*, *SPP1*, *CD68*, *LIPA*, *LGALS3*, *CTSL*, *TGFBI*, *FABP5*, and *CXCL2* (**Figure 7C**), suggesting that P_Mφ2 may represent a conserved macrophage subtype associated with pregnancy, although further studies are needed to confirm this.

To further explore the cross-species conservation and heterogeneity of macrophages in more tissues, we extracted and integrated macrophages from 11 tissues shared by our study and publicly available single-cell profiles of humans (55) and mice (56). Through subcluster analysis, we identified eight macrophage subtypes (Mf1–Mf8) (**Figure 7D**) and assessed their cross-species subtype similarity using MetaNeighbor (54). The P_Mf2 (pig) had a high similarity with H_Mf2 (human) and M_Mf2 (mouse) (**Figure 7E**), with shared high expression of splenic red pulp macrophage marker genes *VCAM1*, *HMOX1*, *CD5L*, and *SPIC* (14) (**Figure 7F**). Likewise, P_Mf4 was highly similar to H_Mf4 and M_Mf4, all characterized by elevated expression of *LGASL1*, *VIM*, and *S100A10* (**Figures 7E, F**). Additionally, P_Mf7 showed high similarity with H_Mf7 and M_Mf7 and highly expressed *ID3*, a marker gene of Kupffer cells in the liver (13) (**Figures 7E, F**). Despite these conserved subtypes, we also observed interspecies heterogeneity. For example, *CPVL*, a serine carboxypeptidase that may be involved in antigen processing, was highly expressed in almost all macrophage subtypes in humans but was hardly expressed in pigs and mice. In contrast, *C4BPA*, which form part of the extracellular complement regulator C4b-binding protein and, a key soluble regulator of the classical complement pathway, was mainly expressed in macrophage subtypes of pigs. Overall, our results suggested a certain degree of conservation among macrophages in humans, pigs, and mice, with this conservation being more pronounced under the same physiological conditions and in specific tissue-resident macrophages.

## 4 Discussion

Macrophages are widely distributed across tissues throughout the body and possess the ability to modulate their phenotype and function to adapt to diverse tissue microenvironments and physiological conditions, playing a central role in immune defense, tissue repair, and maintaining homeostasis. Single-cell technologies have been increasingly applied in pigs to reveal macrophage characteristics under various conditions, including healthy tissue homeostasis and viral infection (36, 81). These studies have provided valuable insights into the dynamic roles of macrophages in maintaining tissue homeostasis and responding to pathological states. Pregnancy represents a unique physiological state during which tissue remodeling and immune adaptations occur to support fetal development, setting it apart from other physiological conditions (18, 82). Here, we constructed a single-cell transcriptome atlas of healthy pregnant pig macrophages comprising nearly 115,000 cells from 49 different tissues/organs of a single individual, providing a rich resource for the in-depth exploration of macrophage biology. Using a single individual for sampling significantly minimizes the impact of the confounding effects associated with genetic background, health status, and other individual-specific factors, which ensures that the observed heterogeneity in macrophage gene expression profiles and functions is largely attributed to tissue-specific microenvironments rather than inter-individual variability (44). Our study identified 33 distinct macrophage subtypes across tissues, except for well-characterized tissue-specific subtypes such as MG in the brain and KCs in the liver, expanding our understanding of tissue-specific macrophage subtypes in other tissues, like mammary gland, lymph node, and kidney. Comparative analysis of cell subtype composition within tissues revealed that the well-known tissue-specific macrophage subtypes (MG, KCs, and RPMs) constituted the core cell populations in their respective tissues and also existed in other tissues in a certain proportion. To our knowledge, a recent study of the human prenatal immune cell atlas also identified and validated peripheral microglia (42). Of note, adipose tissues from different anatomical locations exhibited significant heterogeneity in the composition of macrophage subtypes, underscoring the complexity of immune regulation in fat depots. Our discovery extended a recent report showing that a small number of macrophage subtypes are unique to certain fat depots (83).

Further exploration of the PRRs signaling pathway and scavenger function activity among macrophage subtypes revealed striking differences, particularly in mesenteric adipose tissue and uterus. We observed Mφ MARCO+ in mesenteric adipose tissue exhibited high activity on five PRRs signaling pathways compared with macrophage subtypes in other tissues, including other fat depots. It has been widely acknowledged that the gut has the important task of absorbing nutrients, a complex process that requires an intact barrier that allows the passage of nutrients but simultaneously protects the host from invading microorganisms (84). The mesenteric adipose tissue, situated around the intestinal wall, is adjacent to the gut. This spatial proximity implies that macrophages in mesenteric adipose tissue may be involved in monitoring and responding to microbes and their metabolites in the gut. Therefore, as an immune barrier and “cleaner”, the enhanced PRRs activity of macrophages in mesenteric adipose tissue may contribute to more effectively recognizing and engulfing microorganisms from the intestine to prevent the translocation of gut bacteria (68–70, 85). Our findings highlighted the unique composition and functional characteristics of macrophage subpopulation in different adipose tissues, which may be an adaptation to the unique microenvironment of each site. Our deeply characterized macrophages in adipose tissue provided valuable insights into the complexity of immune cell interactions within various fat depots and the study of metabolic health and disease.

Over the past decade, several molecular determinants that regulate macrophage-specific identity and function have been identified, which are crucial for development, health, neurodegeneration, inflammatory diseases, and tumors (1, 11, 13). Many cytokine family members have been reported to play important roles in pig pregnancy (86). Our study provided a global view of the expression patterns of TFs, cytokines, and cell surface receptors across tissues, all of which are vital for establishing and maintaining the specificity and function of macrophages. We revealed prominent tissue-specific expression of *PLSCR1* in lung macrophages, with GO enrichment analysis indicating its involvement in immune and phagocytosis-related functions. Multiple studies have documented the antiviral properties of *PLSCR1* (87–89), with recent research well-delineating its broad antiviral activity in lung epithelial cells (77). *In vitro* experiments demonstrate that *PLSCR1* expression negatively regulates the FcR-mediated phagocytic activity in differentiated macrophages (90). However, studies on *PLSCR1* in lung macrophages remain limited. Our results suggested that *PLSCR1* may play a key role in enhancing the immune defense capacity of macrophages and speculated that it may be a key factor in regulating macrophages to prevent pathogens from invading the lung environment. Furthermore, the tissue-specific expression patterns observed in cytokines, particularly in the TNF-related family, further emphasized their role in tissue-specific immune modulation. For instance, the tissue-restricted expression of *TNF* and *TNFAIP6* in the lung, and *TNFSF12* and *TNFSF13B* in the epididymis. The chemokine superfamily is grouped into four subfamilies [CXC, CC, (X)C, and CX3C], participating in immune and inflammatory responses, leukocyte migration, and angiogenesis (91). We observed that the members of the CCL and CXCL families were widely expressed in multiple tissues, indicating a common mechanism of macrophage recruitment and migration. These findings collectively revealed the adaptive regulatory network of macrophages in diverse tissue environments, laying the foundation for understanding the role of macrophages in homeostasis and disease states.

During normal pregnancy, transitioning from the pro-inflammatory stage before embryo implantation to the later anti-inflammatory stage, the maternal body undergoes a complex inflammatory regulation that helps maintain maternal-fetal balance. Macrophages play a crucial role in this delicate balance (21, 92, 93). Our comparative analysis of pregnant and non-pregnant uterine macrophages revealed that macrophage subtypes primarily in pregnant sows highly expressed *CD209*, *MRC1*, and *CD163*, implying that macrophages in late pregnancy were polarized into anti-inflammatory M2-like subtypes, which contribute to maternal immune tolerance to the fetus and healthy fetal development (94, 95). It was consistent with the characteristics of human uterine macrophages in the late gestation stage (21, 95, 96), indicating a certain degree of conservatism in the physiological state of pregnancy between humans and pigs. The uterine macrophages of pregnant sows were also accompanied by a significant increase in phagocytic capability. Throughout pregnancy, as the uterus undergoes significant remodeling and expansion to support fetal development, the increased phagocytic capability of macrophages ensures efficient clearance of apoptotic cells, extracellular debris, and harmful immune complexes, thereby preventing inflammatory responses that could jeopardize pregnancy and helping to protect the fetus from maternal immune attack (23, 97, 98). Thus, the increased phagocytic activity of macrophages in late pregnancy is a critical adaptation, supporting maternal and fetal well-being as pregnancy enters its final stages. Moreover, our analysis revealed metabolic reprogramming of uterine macrophages in late pregnancy, with upregulation of genes involved in glycosaminoglycan catabolism, aminoglycan catabolism, and peptide catabolism. These catabolic shifts may reflect the role of uterine macrophages in remodeling the extracellular matrix (99) during late pregnancy, creating an optimal environment for fetal delivery. Overall, the dynamic nature of macrophages during late pregnancy highlighted their adaptability and essential role in supporting the complex physiological changes that occur as the pregnancy approaches term.

As a valuable biomedical model, the pig offers unique insights into human biology (28). Although the type of placenta differs between humans and pigs (32), the striking similarity between the pregnancy-associated macrophage subtype in pigs (P_Mφ2) and human decidual macrophages (H_Mφ3 and H_Mφ8) suggested that certain macrophage programs linked to pregnancy adaption may be conserved across mammals. Moreover, we identified similar macrophage subtypes across pigs, humans, and mice. However, notable species-specific differences, such as the human-specific expression of *CPVL* and pig-specific enrichment of *C4BPA*, may reflect divergent molecular strategies shaped by evolution, or since the downloaded datasets were from non-pregnant individuals, physiological differences may influence the results. Further exploration of conservation and heterogeneity across species will promote our understanding of biological evolution from the perspective of immunity, facilitating translational research among species.

Using a single individual to construct a whole-tissue macrophage atlas offers the advantage of minimizing genetic, epigenetic, and other individual-specific factors that could confound cross-tissue comparisons. However, the sample size remains a limiting factor, and expanding the number of biological replicates is essential to validate and extend our conclusions. Additionally, although we identified several tissue-specific macrophage subtypes, their potential functions remain to be elucidated through further experimental validation. Finally, extending the atlas to include more tissues from non-pregnant individuals could provide deeper insights into how macrophages in various tissues adapt to the physiological demands of pregnancy, complementing the framework established in this work.

## 5 Conclusion

Taken together, we constructed a multiple-tissue macrophage single-cell transcriptome atlas of the pregnant pig, shedding light on the heterogeneity of macrophage subtype composition and gene expression profiles under different tissue microenvironments, alongside a preliminary exploration of the adaptive functional changes of uterine macrophage subtypes in pregnancy. Additionally, cross-species analysis further highlights the conservation of macrophage subtypes. This work deepens our understanding of macrophage biology during pregnancy and provides a valuable resource for exploring macrophage diversity and tissue-specific macrophage adaptations during pregnancy in pigs.

## Data Availability

The datasets generated in the current study are available from the Genome Sequence Archive (GSA) database (accession number CRA021679) and FigShare (DOI: 10.6084/m9.figshare.28588511). The original analysis code in this paper has been deposited at Zenodo (https://doi.org/10.5281/zenodo.15010409).
