# Supplementary material for: Cross one single body 49 tissues single-cell transcriptome reveals detailed macrophage heterogeneity during pig pregnancy
Source: Front Immunol. 2025 Apr 2;16:1574120. doi: 10.3389/fimmu.2025.1574120 (PMC12000058; doi:10.3389/fimmu.2025.1574120)
Supplement: Supplementary file 1 [file DataSheet1.pdf]

Supplementary Figures

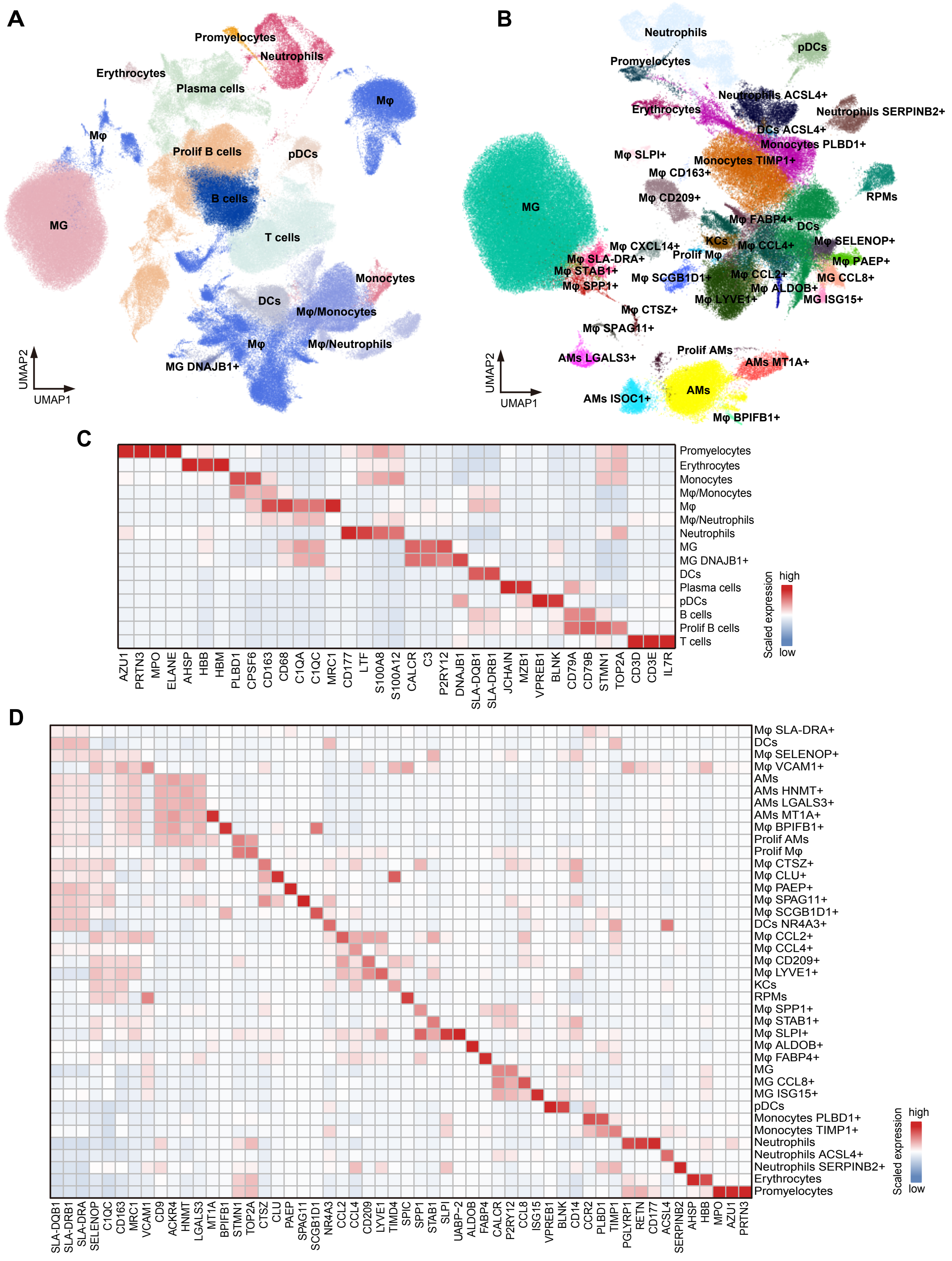

Supplementary Figure 1. Cross-tissue single-cell transcriptome atlas of pig immune and myeloid cells.

(A) UMAP visualization of immune cells (249,689 cells) colored by cell types. **(B)** UMAP visualization of myeloid cells (164,113 cells) colored by cell types. **(C)** Heatmap depicting the expression of manually selected marker genes for all identified immune cell subtypes in (A). **(D)** Same visualization as (C), but for myeloid cells identified in (B). Color scale: red, high expression; blue, low expression. Mφ, macrophages; MG, microglia; DCs, dendritic cells; pDCs, plasmacytoid dendritic cells; Prolif, proliferating; AMs, alveolar macrophages; KCs, Kupffer cells; RPMs, red pulp macrophages.

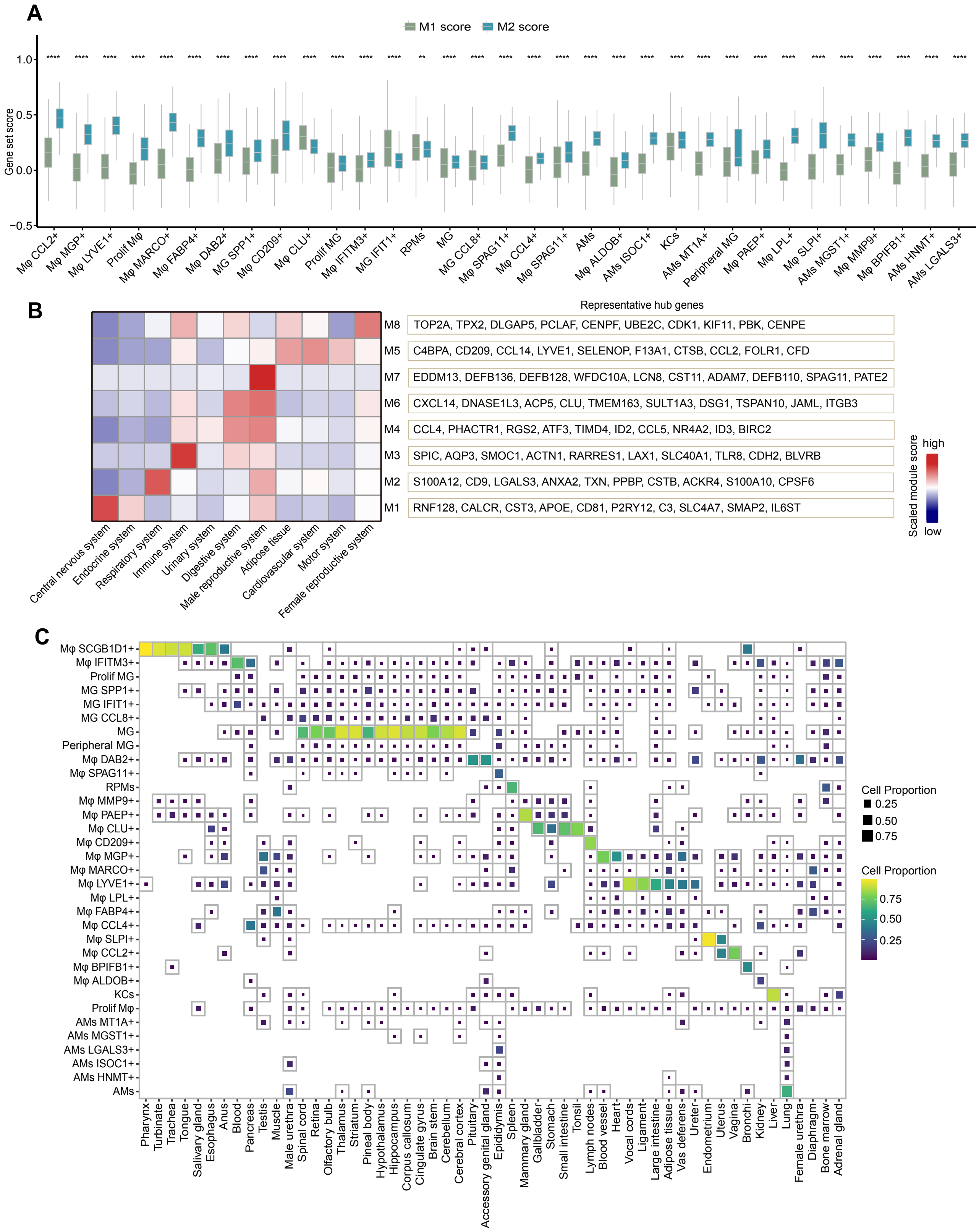

Supplementary Figure 2. Functional heterogeneity and tissue distribution characteristics of macrophage subtypes.

(A) Difference in gene set scores of M1 and M2 macrophage polarization-related genes among macrophage subtypes. The significant differences between M1 and M2 scores were marked by asterisks (Wilcoxon rank sum test, * $p < 0.05$; ** $p < 0.01$; *** $p < 0.001$; **** $p < 0.0001$). **(B)** The heatmap of macrophage gene module scores of 11 systems. Representative hub genes of the eight modules are shown on the right. Color scale: red, high score; blue, low score. **(C)** The composition of macrophage subtypes in different tissues/organs. Both size and color indicate the cell proportion of each macrophage subtype in the respective tissues/organs.

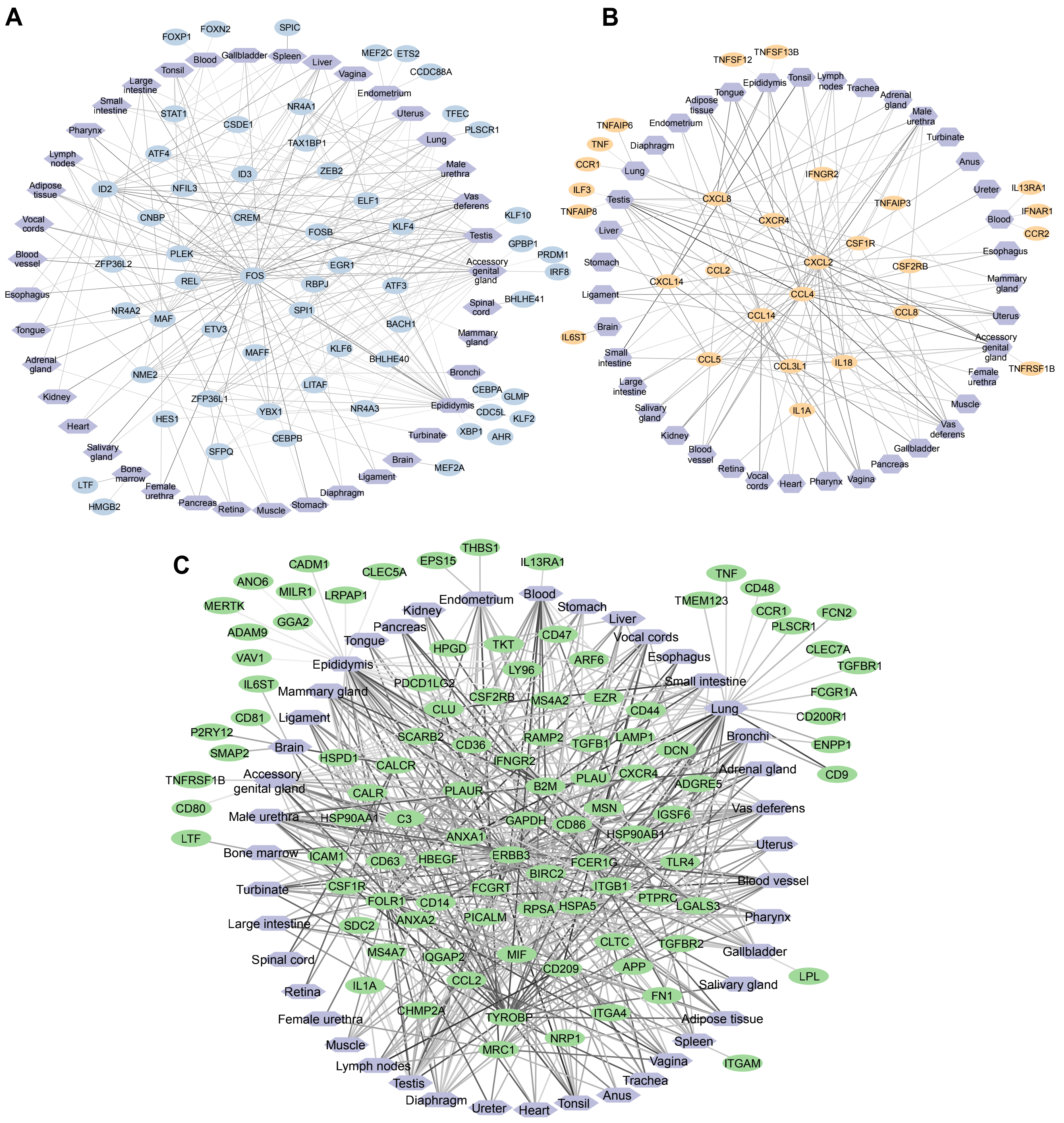

Supplementary Figure 3. Visualization of the cross-tissue distribution and expression of differentially expressed TFs, cytokines, and cell surface receptors identified in each tissue/organ.

(A) Network of the expression patterns between TFs and the tissues/organs. **(B)** Network of the expression patterns between cytokines and the tissues/organs. **(C)** Network of the expression patterns between cell surface receptors and the tissues/organs. In each panel, purple nodes indicate tissues, blue nodes indicate TFs, orange nodes indicate cytokines, and green nodes indicate cell surface receptors. The edges between nodes represent gene expression levels, with darker colored edges corresponding to higher gene expression in the corresponding tissue.

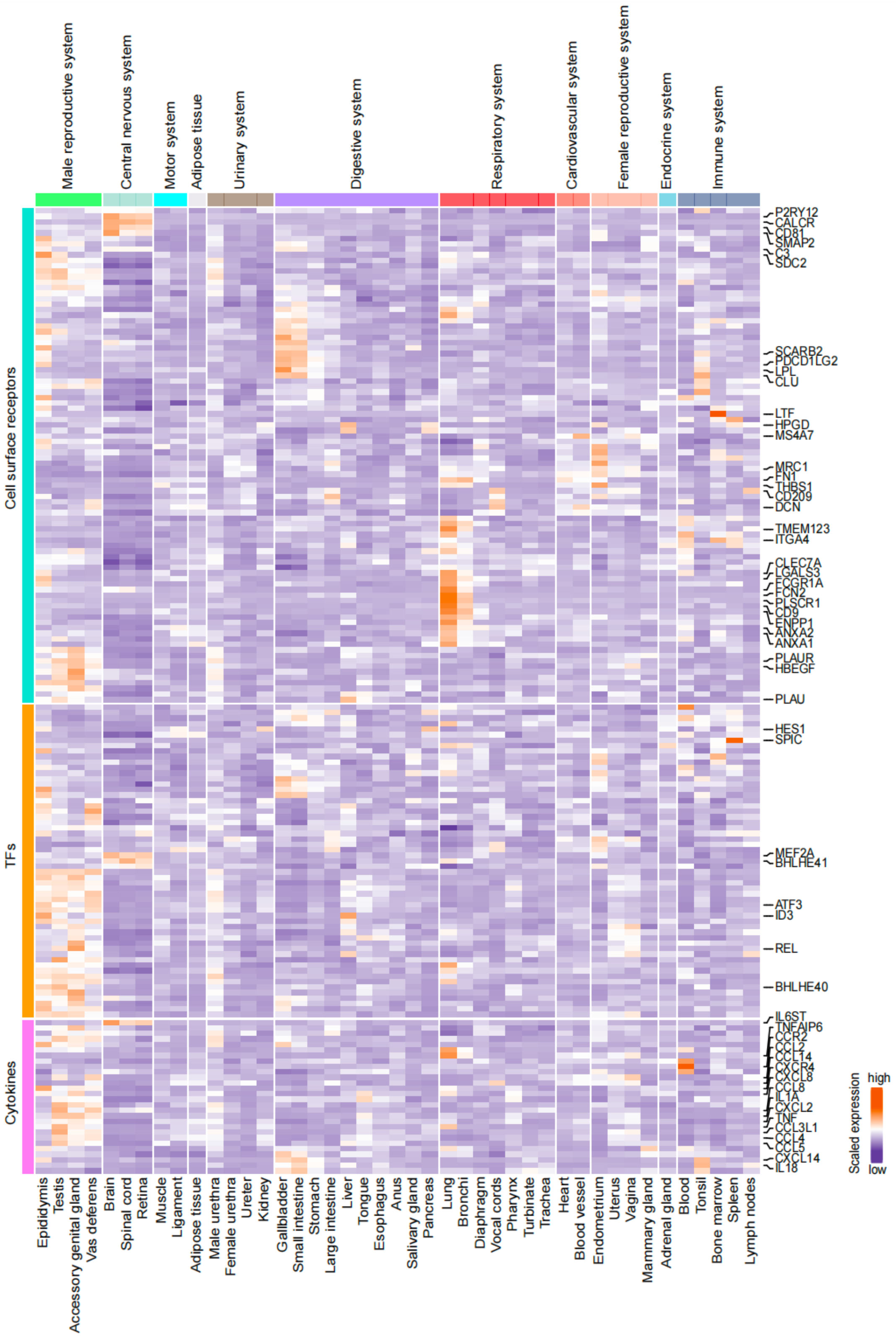

Supplementary Figure 4. Heatmap showing the global expression patterns of differentially expressed TFs, cytokines, and cell surface receptors identified in each tissue/organ.

The bar above represents the system, and the bar on the left represents the type of gene. The marked genes are tissue-specific high expression genes (only identified in one tissue).
